# Perceptual switch rates with ambiguous structure-from-motion figures in bipolar disorder

**DOI:** 10.1098/rspb.2008.0043

**Published:** 2008-05-07

**Authors:** Kristine Krug, Emma Brunskill, Antonina Scarna, Guy M Goodwin, Andrew J Parker

**Affiliations:** 1 Department of Physiology, Anatomy and Genetics, University of OxfordOxford OX1 3PT, UK; 2 Department of Psychiatry, Warneford Hospital, University of OxfordOxford OX3 7JX, UK

**Keywords:** decision making, rivalry, perception, extrastriate visual cortex, bistable figures, visual neuroscience

## Abstract

Slowing of the rate at which a rivalrous percept switches from one configuration to another has been suggested as a potential trait marker for bipolar disorder. We measured perceptual alternations for a bistable, rotating, structure-from-motion cylinder in bipolar and control participants. In a control task, binocular depth rendered the direction of cylinder rotation unambiguous to monitor participants' performance and attention during the experimental task. A particular direction of rotation was perceptually stable, on average, for 33.5 s in participants without psychiatric diagnosis. Euthymic, bipolar participants showed a slightly slower rate of switching between the two percepts (percept duration 42.3 s). Under a parametric analysis of the best-fitting model for individual participants, this difference was statistically significant. However, the variability within groups was high, so this difference in average switch rates was not big enough to serve as a trait marker for bipolar disorder. We also found that low-level visual capacities, such as stereo threshold, influence perceptual switch rates. We suggest that there is no single brain location responsible for perceptual switching in all different ambiguous figures and that perceptual switching is generated by the actions of local cortical circuitry.

## 1. Introduction

Ambiguous images that produce more than one coherent percept (such as the Necker Cube, Rubin's Face/Vase Image, structure-from-motion (SFM) and binocular rivalry) are a major tool for investigating the neural basis of perception (Blake & Logothetis 2002; Parker & Krug 2003). When humans view such stimuli, typically the percept flips unpredictably between one appearance and the other. This perceptual switch is interesting, because the physical stimulus stays constant while the percept alternates (Wheatstone 1838; Blake & Logothetis 2002).

There is considerable debate about the brain signal that drives the switch. In the case of binocular rivalry, researchers have argued for at least three possibilities: first, a low-level ‘eye-driven’ mechanism at the level of primary visual cortex (V1); second, a higher cortical mechanism of pattern suppression; and third, an interhemispheric switch (for recent accounts see: Sengpiel *et al*. 1995; Miller *et al*. 2000; Polonsky *et al*. 2000; Blake & Logothetis 2002). Neuronal activity in extrastriate areas, such as V5/MT or inferotemporal cortex, modulates with the dominant percept in binocular rivalry (Logothetis & Schall 1989; Sheinberg & Logothetis 1997) and also to a lesser extent in V1 (Leopold & Logothetis 1996; but see Lee *et al*. 2005).

The rate of perceptual switching in binocular rivalry has been investigated in bipolar disorder by Pettigrew & Miller (1998) who concluded that the intervals between switches were longer in bipolar participants than controls. Other studies have also found slower switch rates for ambiguous figures in bipolar patients (Hunt & Guildford 1933; Miller *et al*. 2003). Slowing of switch rates was termed a ‘sticky switch’ by Pettigrew & Miller (1998), a concept that has generated substantial theoretical and practical interest.

If there were a sticky switch, located at a single brain site at a high level of visual processing, then slower switch rates in bipolar patients should be a general feature of the response to ambiguous figures of all kinds (Carter & Pettigrew 2003; Meng & Tong 2004; van Ee 2005). We investigated whether this difference in switch rates between bipolar patients and controls was present for a different ambiguous figure, namely a cylinder defined by SFM whose direction of rotation is bistable (Wallach & O'Connell 1953; Treue *et al*. 1991).

SFM stimuli offer several advantages for this kind of study in comparison with binocular rivalry (Parker & Krug 2003). First, perceptual switches are complete, with hardly any intermediate percepts, unlike binocular rivalry (e.g. Wilson *et al*. 2001). Second, separating the front and back surfaces of the cylinder with binocular depth disambiguates its direction of rotation. Ambiguous SFM figures are indistinguishable from unambiguously rotating figures with small binocular disparity (Nawrot & Blake 1993). In this study, we exploited this last point to check the attention and performance of any participant that we tested. Last, neurophysiological recordings in the awake macaque indicate a potential mechanism for perceptual switching (Parker & Krug 2003). Neuronal signals in the V5/MT area correlate with the reported percept for this bistable stimulus (Bradley *et al*. 1998; Dodd *et al*. 2001). Unlike the case with binocular rivalry (Logothetis & Schall 1989), the neuron's direction of rotation associated with perceptual reports about ambiguous cylinders is always concordant with the direction of rotation preferred for unambiguous cylinders (Dodd *et al*. 2001; Krug *et al*. 2004). Thus, the bistability of the percept for the cylinder stimulus is thought to be directly due to the competition between neuronal populations in V5/MT, which can be identified directly from the selectivity of those neurons for unambiguous stimuli.

## 2. Material and methods

### (a) Participants

Twenty-five bipolar patients (11 females, 14 males), aged 24–77 years (mean=40.9 years), were recruited. Consultant psychiatrists had previously diagnosed all patients with bipolar disorder (DSM-IV). Each patient was screened for recent mood disorder and current psychiatric co-morbidity before experimental data were collected. This eliminated two patients (one for alcoholism and the other for obsessive compulsive disorder). Participants' stereovision was tested (see §2*c*) and three patients were excluded because their stereo threshold was 0.5° or higher. The study group comprised 20 bipolar patients: 11 females, 9 males; age range 24–77 years; and mean age 42.8 years.

Twenty-five control participants (13 females, 12 males; age range 20–66 years; mean age 32.8 years) were recruited from Oxford area residents and University faculty and students. They were screened using a Structural Clinical Interview from DSM-IV (Diagnostic and Statistical Manual of Mental Disorders) Axis 1 test for previous psychiatric history: only participants without Axis 1 psychiatric diagnoses were included for the baseline data. Three control participants were excluded owing to their inability to identify stereo cues (see above). The control group comprised 22 participants: 12 females, 10 males; age range 20–58 years; and mean age 31.0 years. The participants included 18 naive and 4 experienced participants. There was no significant difference between the mean percept durations of the naive and experienced participants (Mann–Whitney test, *p*=0.22).

On the day of testing, patients were assessed on the Young Mania Rating Scale (Young *et al*. 1978; *n*=20, mean score=2.8, range=0–10), the Hamilton Depression Scale (Hamilton 1960; *n*=20, mean=3.2, range=0–8) and the Beck Depression Inventory (Beck *et al*. 1961; *n*=20, mean=6.7, range=0–16.5). All patients were clinically euthymic (neither manic nor depressed) around the time of testing. All participants had normal or corrected to normal vision, gave informed consent in writing and were paid for their participation. The study was approved by the Oxfordshire Psychiatric Research Ethics Committee.

### (b) Visual stimuli

Stimuli were displayed using either a Wheatstone stereoscope in the Department of Physiology, Anatomy and Genetics or an anaglyphic method on a single monitor at the Warneford Hospital (see the electronic supplementary material). Experiments were conducted in two locations in order to facilitate the participation of the patient group. The smaller and more portable anaglyph set-up was used at the hospital site. For the anaglyph set-up (Warneford), stimuli were displayed on a Viglen ENVY 17Si monitor 100 cm from the observer; the stereoscope (Physiology) comprised two EIZO FlexScan T961 monitors 265 cm from the observer. The stimulus was made up of white dots on a grey background and was always presented in the centre of the screen (refresh rate 60 Hz). Dots subtended 0.063×0.063° (Warneford) or 0.044×0.044° (Physiology). The dot density was 5.6 dots deg^−2^ of visual angle (Warneford) or 26.17 dots deg^−2^ of visual angle (Physiology). The stimulus subtended 6° (Warneford) or 4.5° (Physiology) of visual angle. The angular rotation velocity of the cylinder about its principal axis was 90° s^−1^ (4 s for a complete rotation) in both the set-ups.

The difference in dot density between the two set-ups is not ideal, because increasing dot density in a rotating SFM sphere reportedly decreases the perceptual duration (Brouwer & van Ee 2006). However, any decrease in percept duration for those control participants tested on the Physiology set-up would actually heighten the predicted difference between bipolar and control participants—counter to what we report in this paper. Furthermore, by dividing the controls between both sets of equipment, we established that there were no significant differences in perceptual durations in our study (see the electronic supplementary material).

**Figure 1:**
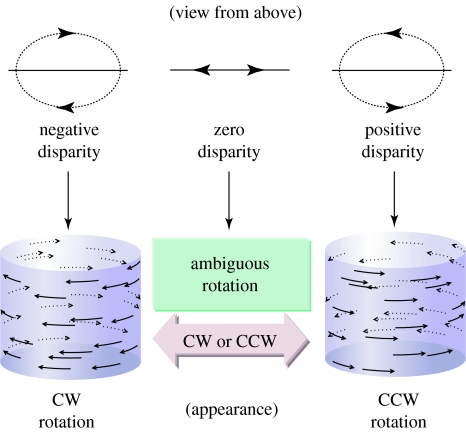
SFM cylinders were constructed from two transparent sheets of random dots moving in opposite directions. When dots were at the same depth (binocular disparity=0), the direction of rotation was bistable. Binocular disparities applied to the left- and rightward moving dots disambiguate the direction of cylinder rotation: putting the leftwards moving dots in front and the rightwards moving dots at the back yields a clockwise rotating cylinder (as viewed from above). CW, clockwise; CCW, counter clockwise. Reprinted with permission from Parker & Krug (2003, p. 434). Copyright © Elsevier.

Participants viewed a cylinder composed of two transparent planes of random dots moving in opposite directions (to the left and right). The cylinder's axis of rotation was always vertical. Except for catch events (see below), all dots had a binocular disparity of 0°. At stimulus onset, each dot was placed at a random location and then moved with a sinusoidal velocity profile, as would be expected in a two-dimensional projection of a rotating three-dimensional cylinder. To prevent participants from tracking individual dots, on each video frame 1% of the dots were replaced by new dots at random locations within the cylinder (median dot lifetime 1.15 s). See figure 1 and the electronic supplementary material for stimulus illustrations and further discussions about its perceptual appearance.

### (c) Experimental protocol

In order to estimate each participant's stereo threshold before data collection, they were shown unambiguous cylinders, whose direction of rotation was specified by binocular disparity (3 s duration; 16 trials at each disparity; clockwise and anticlockwise rotation randomly interleaved). Participants reported the direction of cylinder rotation by pressing a keyboard button. If participants were unsure, they were asked to make a best guess. Binocular disparities of 0.025, 0.05, 0.075, 0.1, 0.2, 0.4 and 0.5° were used. The lowest disparity at which a participant judged all 16 trials correct was selected as the stimulus for the catch events (see below) and was also used as an estimate of the stereo threshold for further analysis. If a participant could not reliably judge the direction of rotation at the largest disparity tested (0.5°), they were excluded from the study. This preliminary screening allowed us to be confident that, in principle, participants could report the stimulus correctly.

For the experiment, each trial consisted of a 4 min period of continuous viewing of a cylinder stimulus. All patients and naive controls were told that the cylinder would occasionally reverse its direction of rotation. We were careful not to tell them that the stimulus was ambiguous. They were asked to indicate the initial direction of rotation of the cylinder and then to indicate the new direction of rotation every time the direction changed. Participants were instructed to correct their response, if they felt they had made a mistake. There was a break of 3 min every three trials.

In order to monitor participants' attention to the task, we included ‘catch events’ during the viewing of the ambiguous figure. Catch events were brief periods (9 s) when binocular disparity was added to the display so that the cylinder was rotating unambiguously. The added disparity was above each individual's stereo threshold, so if participants are paying close attention, they should respond accurately to this unambiguous rotation. A correct response to a catch event required identification of the unambiguous direction of rotation during the 9 s of introduced binocular disparity. The introduced unambiguous cylinder was concordant with the current percept in 50% of cases (i.e. participants should not switch percept), but in the other 50% of cases, they had to respond to the change in direction of rotation.

These catch events provide a measure of the participants' level of attention to the task without inducing a bias on the reporting of any particular direction of rotation. Participants who correctly identified 75% or more of the catch events were defined to be attending to the task. This provides confirmation that they were faithfully responding to changes in the perceived direction of rotation, neither failing to detect changes that did occur nor falsely reporting changes that did not occur.

In order not to interfere with the reporting of spontaneous reversals of the ambiguous cylinder, catch events were randomly generated 15% of the time anywhere between 0.5 and 5 s following a key press reporting that an ambiguous stimulus had changed direction of rotation. This was lowered to 10% for a few participants who were switching very frequently, because enough catch events were being generated.

Since catch events are not commonly used in studies of ambiguous figures, it is a reasonable concern that they could affect percept durations. It is already known that unambiguous SFM stimuli can influence the perception of subsequent ambiguous stimuli (Nawrot & Blake 1991). Therefore, we excluded responses (and corresponding percept durations) immediately following catch events (see §2*d*).

Before data collection, participants undertook a practice session, which consisted of three or four 4 min trials; each trial was followed by a 1 min break. The practice trials were intended to ensure that participants were familiar with the task and that their switch rates had stabilized. Data from the practice sessions were not included in the analysis.

During practice sessions, some participants appeared to be stuck in one percept and did not appear to flip over to the alternate percept. This finding is consistent with earlier work, which showed that, for some ambiguous figures, naive participants might only see one perceptual interpretation, if they did not already know that the stimulus was bistable (Rock & Mitchener 1992). Such participants need the alternative percept to be explicitly pointed out before they start to switch (Leeper 1935; Attneave 1971). Therefore, for these participants, extra catch events were induced during practice trials, frequently creating a percept opposite to the one currently perceived. In general, participants then started to self-generate switches during ambiguous stimulus presentation.

After the practice session, most participants performed a full run of 12 trials as described above (total viewing time 48 min). For eight participants (three controls, five bipolar patients), only a smaller number of trials could be completed (six to ten trials, 24–40 min viewing time). The participants were encouraged to request extra breaks as needed, and many participants took a longer break half way through the full run.

### (d) Data analysis

Percept duration is the time for which a participant views the cylinder as rotating in one direction. We ignored responses that reported the same direction of rotation as the immediately preceding response as well as responses (and corresponding percept durations) during catch events (see above). We excluded percept durations truncated by catch events and responses (and corresponding percept durations) immediately following catch events. We analysed the duration between the first key press after a catch trial and the next key press (first subsequent full duration). No controls and only one patient showed a significant difference in percept durations immediately following a catch trial (Mann–Whitney test). Therefore, these durations were treated similarly to the other durations in our analysis.

We also excluded all durations less than 250 ms, along with the percept durations on either side, since these are probably due to participants accidentally pressing two keys simultaneously or in short sequence. Indeed, previous work has shown that reaction times for detecting SFM are approximately 1 s long (Treue *et al*. 1991). We also removed all percept durations truncated by the end of trial, except for trials where only one direction of rotation was recorded. The complete removal of all percept durations truncated by the end of the trial did not alter our conclusions.

Matlab was used to analyse the data. The percept durations for individual participants were not normally distributed. In order to enable the use of parametric statistical tests, participants' data were log transformed (transformed data=log(percept durations)): the resulting data were approximately normally distributed. Several distributions (gamma, lognormal and gamma rate for the switch rates) were fitted to the data, using the ‘gamfit’ and ‘lognfit’ routines in the Matlab statistics toolbox. The goodness of fit of each distribution to the data was tested using a *Χ*^2^-test.

We applied a likelihood ratio test to examine whether the pooled percept durations for the two groups could be fit with the same set of lognormal distribution parameters, or whether a significantly better fit could be obtained by allowing each group to be separately fit. A simple transformation of the likelihood ratio (−2×log(ratio)) is distributed as a *Χ*^2^-distribution with two degrees of freedom.

## 3. Results

### (a) Perceptual stability of the SFM cylinder

**Figure 2:**
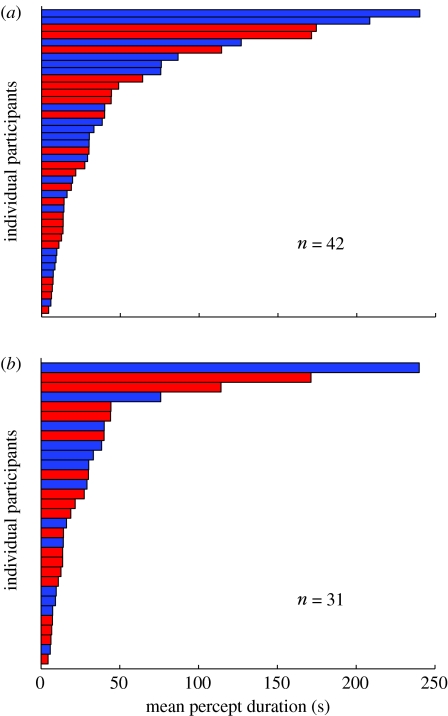
The distribution of mean percept durations for (*a*) all participants and for (*b*) participants who scored greater than or equal to 75% correct for catch events. Red bars depict control participants and blue bars the participants with bipolar disorder. There was no obvious segregation between the percept durations from the two participant groups.

We measured the rate at which participants reported reversals in the cylinder percept during long periods of continuous viewing (trials of 4 min duration). Across all 22 control participants, the raw data showed a mean percept duration of 41.1 s (see figure 2*a*, red bars for distribution of individual percept durations).

The accurate psychophysical measures of the timing of perceptual reversals depend crucially on the cooperation and attention of the participants. It is difficult to apply objective performance criteria that do not interfere with the actual measurements. Therefore, we included catch events in some trials (see §2). Control participants responded correctly to 89% of catch events (averaged across all control participants). Most errors arose when participants failed to detect a switch in perception implied by the introduction of an unambiguous disparity inconsistent with the current percept (82.3% of errors across all control participants). The remaining errors arose when participants falsely reported a switch when the added disparity was consistent with the currently reported percept. Errors of this kind were rare, occurring on only 2% of catch trials altogether. When we constrained the analysis for control participants to those who responded correctly to at least 75% of catch events (*n*=18), the mean percept duration was reduced from 41.1 to 33.5 s. Nonetheless, average percept durations still differed widely between control participants, ranging from 4.5 to 171.4 s (figure 2*b*; red bars).

In comparison with other bistable figures, the mean switch rate (0.03 Hz) for changing the perceived direction of rotation was low. The percept for the ambiguous SFM cylinder appeared more stable than the percept for other bistable figures, especially binocular rivalry (e.g. Brascamp *et al*. 2005).

### (b) Perceptual switch rates for bipolar patients

We compared the mean percept duration for the cylinder between 20 bipolar patients (11 females, 9 males; 24–77 years (mean 42.8)) and 22 controls (12 females, 10 males; 20–58 years (mean 31.0)). For the bipolar patients, the rotating cylinder appeared to change its direction of rotation on average every 55.3 s, longer than the 41.1 s for the control participants. The difference was not statistically significant (Mann–Whitney test, *p*>0.42) and the distributions overlapped extensively (figure 2*a*).

Many bipolar patients correctly responded to 75% of catch events. Like the controls, the majority of errors by patients consisted of missing a perceptual switch indicated by a catch event (71.9% of errors). When we restricted the analysis to participants who detected 75% or more of the catch events, the mean percept duration for bipolar patients reduced to 42.3 s (*n*=13; compared with 33.5 s for the controls). Again, the difference between bipolar patients and controls was not statistically significant (Mann–Whitney test, *p*>0.63). The observed switch rates for those bipolar patients who reliably detected the catch events ranged from 0.0042 to 0.1693 Hz. Again, there was an extensive overlap between the measurements from the two participant groups (figure 2*b*). It would be impossible to identify individuals with bipolar disorder based on this test. Therefore, switch rates for bistable SFM figures appear unsuitable for serving as a trait marker for bipolar disorder.

### (c) A more subtle difference between bipolar and control groups

We were concerned that we may have missed a large difference between the bipolar and control groups of the kind previously reported for binocular rivalry (Pettigrew & Miller 1998), so we checked the statistical power of our experiment. Pettigrew & Miller (1998) reported a *z*-score of 4.56 (derived from the non-parametric Mann–Whitney test). Even allowing for the small inefficiency of the non-parametric test, for a two-tailed test with *α* set at 0.05, the statistical power for comparing mean switch rates for all participants (controls, *n*=22; bipolars, *n*=20) is in excess of 0.99. Therefore, with our experimental design, we may reasonably expect to detect a difference in perceptual durations as big as that reported for binocular rivalry.

Percept durations for ambiguous figures do not usually follow a normal distribution (Borsellino *et al*. 1972; Logothetis *et al*. 1996; Brascamp *et al*. 2005). Therefore, we initially relied on non-parametric statistical tests (Mann–Whitney test) for the analysis of differences in percept durations and switch rates. Such tests are inherently more conservative than a parametric test. To explore whether we could properly use a parametric test, we examined various transformations of the participants' percept durations. In other similar studies, the gamma distribution (Borsellino *et al*. 1972; Logothetis *et al*. 1996), the gamma rate distribution (Brascamp *et al*. 2005) or the lognormal distribution (Zhou *et al*. 2004) have been variously advocated. However, in some studies, there was no attempt to establish whether the data satisfied a test for the goodness of fit; in others, the data were trimmed to remove the longest and shortest percept durations before fitting, without a clear rationale for doing so.

**Figure 3:**
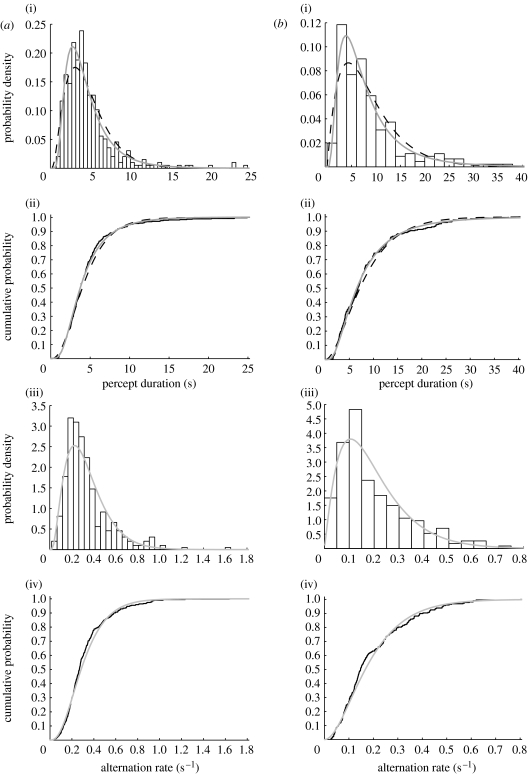
(*a*) Individual percept durations for one control participant (mean percept duration 4.53 s). We fitted a gamma distribution and a lognormal distribution to the percept durations: (i) the probability density function (PDF; bars, data; black dashed lines, gamma fit; grey solid lines, lognormal fit); (ii) the cumulative distribution functions (CDFs) with the best-fitting gamma and lognormal distributions (black solid line, data; black dashed lines, gamma fit; grey solid line, lognormal fit); (iii) PDF (bars, rates; grey solid lines, gamma rate fit) and (iv) CDF of the participant's switch rates (inverse percept durations) with best-fitting gamma rate distribution (black solid line, rates; grey solid line, gamma rate fit). The lognormal fit appeared to provide the best fit for this individual. (*b*) As in (*a*), for a bipolar participant (mean percept duration 8.52 s). On visual inspection, the best fit appeared again to be provided by the lognormal distribution.

We fitted the percept durations for each participant with a gamma, gamma rate and lognormal distributions; examples are shown in figure 3. On informal examination, the lognormal distribution seemed to provide the best fit. We pursued this qualitative insight with two quantitative analyses. First, we pooled normalized percept durations for all the control participants (*n*=22) and for all bipolar patients (*n*=20) separately and fitted them again with the three distributions. Statistical analysis (*Χ*^2^) did not fully support any of the three candidate distributions, for either patients or controls. Second, we analysed which distribution best fitted each individual's data. Here, the lognormal distribution provided the best fit (as evaluated by *Χ*^2^ goodness of fit) for the largest number of participants (patients and controls combined; see the electronic supplementary material), in agreement with some previous studies. Therefore, we used the lognormal transformation for further analysis.

**Figure 4:**
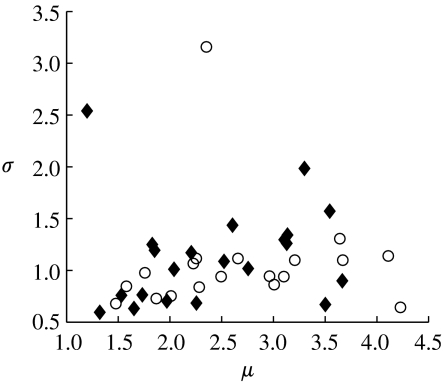
Percept durations were fitted separately for each participant with the best-fitting lognormal distribution. The two fitting parameters for each individual, mean value of the log-transformed data, *μ*, and the square root of variance of the log-transformed data, *σ*, are plotted against each other. Open circles, bipolar participants; closed diamonds, controls. There is no segregation of the parameters between the two groups.

Even where median percept durations are similar, differences in the underlying distribution of perceptual durations may be detectable by analysing the parameters of the fitted distribution (van Ee *et al*. 2006). Therefore, we examined whether there were any differences between the parameters of the best-fitting lognormal distributions for individual controls and patients (figure 4). A Mann–Whitney test (selected to avoid parametric assumptions about the fit parameter distributions) found no statistical difference between the two sets of fit parameters for the two groups (*p*>0.1; see the electronic supplementary material for equivalent results for gamma and gamma rate distributions). Ideally, this procedure requires data distributions with a large number (greater than 100) of percept durations for each participant, but the switch rate of some individuals was too low to achieve this. The exclusion of datasets with less than 100 samples did not alter the conclusions, nor did restricting participants to those who passed the catch events. Since none of these basic tests revealed statistical differences between individuals' fit parameters, we decided to not pursue this line of analysis further.

**Figure 5:**
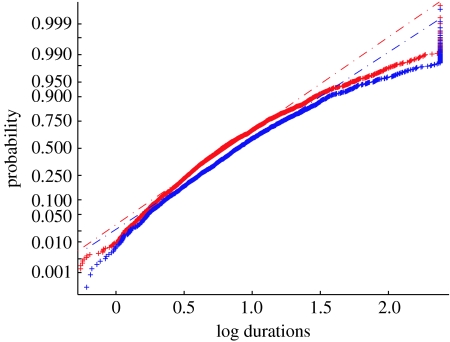
The CDF for all percept durations plotted on a log scale. Patient log percept durations (blue plus symbols) and control log percept durations (red plus symbols) were pooled separately after normalization. Both datasets roughly follow a straight line, indicating that after transformation, the data are approximately normally distributed. The curve for the control participants rises faster indicating that the control group has shorter percept durations than the patient group. The best-fitting line for the control data (red dashed line) could not adequately describe the patient data (blue dashed line; *Χ*_2_^2^=57, *p*<0.001).

Lastly, we adopted a population-level analysis: we pooled percept durations for all control participants (*n*=22) and all bipolar patients (*n*=20) and summarized each group by a best-fitting lognormal distribution. In a lognormal probability plot of the percept durations, the data fall close to the theoretical straight lines, which indicate that the lognormal distribution was close to correct for these data (figure 5). This procedure reveals a small, but highly significant difference between the bipolar patients and the control group: the lognormal distribution that best fits the data for the control participants is inadequate as a fit to the data for the bipolar patients (likelihood ratio test, *Χ*_2_^2^=57, *p*<0.001). This is also true if one includes only the participants who pass the criterion of at least 75% correct detection of catch events (*Χ*_2_^2^=37, *p*<0.001; see the electronic supplementary material). Therefore, the combination of a parametric test and pooling of the data across participants revealed a small, but statistically significant, difference at the level of the two participant groups that is consistent with earlier work.

Results with the bistable SFM cylinder appear to point to a much smaller difference between bipolar and control participants in comparison with previous reports that used binocular rivalry and other bistable images. This could be a genuine difference in the way the brain treats these two types of ambiguous stimuli, but our study also differs from previous studies in the design of the experiments. For example, the fact that we monitored participants' performance on unambiguous SFM during the experiment might be a factor, as might be the fact that we applied stringent screening tests based on visual performance for inclusion in the study.

### (d) Other factors that influence the duration between perceptual switches

**Figure 6:**
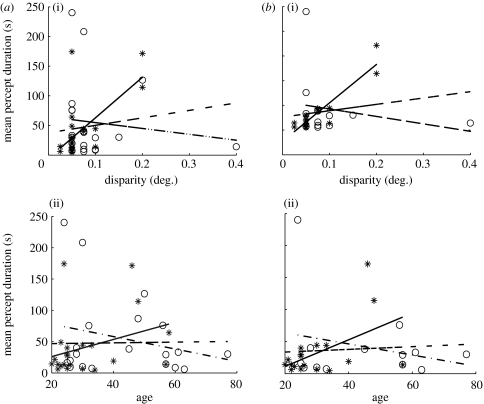
Linear regression of mean percept duration and participants' stereo threshold or age. (*a*) For all participants, the mean percept duration in the control group (asterisks) was significantly correlated with (i) disparity threshold: controls, *r*=0.64, *p*<0.01, *n*=20 (bipolars, *p*=0.61; all, *p*=0.36), but not with (ii) age: controls, *p*=0.15 (bipolars, *p*=0.30; all, *p*=0.92). There was no correlation in the patient group (open circles). (*b*) As in (*a*) for participants with catch events correct greater than or equal to 75%. The mean percept duration for control participants (asterisks) was correlated with (i) disparity threshold: controls, *r*=0.92, *p*<0.001, *n*=18 (bipolars, *p*=0.46; all, *p*=0.33), and (ii) age: controls, *r*=0.51, *p*<0.05, *n*=18 (bipolars, *p*=0.41; all, *p*=0.73). There was no significant correlation for the patients (open circles). Asterisks, control experimental data; solid line, control prediction; open circles, bipolar experimental data; dot-dashed line, bipolar prediction; dashed line, all data prediction.

Previous work suggested that percept durations for ambiguous figures increase with participants' age (Ukai *et al*. 2003). When we examined the relationship between the average percept duration and age or stereo threshold for all control participants, we found a significant correlation for percept duration with stereo threshold but not with age (figure 6*a*). For control participants who detected at least 75% of the catch events, both disparity threshold and age correlated significantly with percept durations (figure 6*b*). Thus, when stereo thresholds were high, switch rates slowed down. Stereo thresholds deteriorate with age, particularly for participants over 50 (Zaroff *et al*. 2003). This is confirmed in our data by a significant linear regression of log(stereo threshold) on age (*p*=0.002) with patient versus control as a grouping factor. Our study only included a small number of participants over the age of 50. Therefore, we expect that the trend of slower switch rates with age might have become significant for the entire control group, if a larger number of older participants had been included.

All participants with a diagnosis of bipolar disorder were euthymic at the time of testing. This was confirmed through the Hamilton Depression Rating Scale, Young Mania Rating Scale and the Beck's Depression Inventory, but low-level symptoms might have contributed to any effect. However, when we compared individual test scores on these scales against the mean percept duration, no significant correlations were found. We also examined the sequence of responses made by the bipolar patients by analysing the temporal autocorrelation function and perceptual bias of the patients' responses in comparison with the control group: there were no significant differences (see the electronic supplementary material).

## 4. Discussion

Previous studies have reported large differences between bipolar and control participants in switch rates for rivalrous and other ambiguous figures (Hunt & Guildford 1933; Pettigrew & Miller 1998; Miller *et al*. 2003). Under conditions in which we carefully monitored attention and performance during the task, we found a small lengthening of perceptual durations in bipolar patients. However, switch rates for ambiguous SFM figures are unlikely to be useful as a trait marker or endophenotype for bipolar disorder. Our results also suggest that perceptual changes of ambiguous figures are not governed by a single, central switching mechanism; otherwise, the differences in the switch rate between the two groups for SFM would be very similar to those found for binocular rivalry.

### (a) Comparison with binocular rivalry

A possible reconciliation of the difference in results for the cylinder and binocular rivalry would be to argue that different brain mechanisms are responsible for ambiguous SFM figures, as opposed to other ambiguous and rivalrous figures (George 1936; Meng & Tong 2004; van Ee 2005). Certainly with binocular rivalry, the perceptual task for the brain is different from that of other ambiguous figures. Rivalry results in the suppression of one out of the two incompatible stimuli in order to arrive at a coherent percept. Both possible percepts are equally acceptable but the temporary dominance of one percept leaves a physical stimulus present before the visual system, which must be suppressed to assist in the perceptual interpretation of the dominant figure (Attneave 1971). This suppression is not an all-or-nothing event. Rivalry can lead to patchy percepts, incorporating segments of both images (Wilson *et al*. 2001).

By contrast, with ambiguous figures such as the Necker cube or the rotating cylinder, the dominant perceptual interpretation fully accounts for the physical stimuli presented to the visual system. When this type of ambiguous figure takes on one particular interpretation, all of the luminance features are completely consistent with whichever interpretation has been adopted. Instead of having to suppress part of the stimulus, the system behaves as if it adds a missing cue, with the effect of disambiguating the image. These differences in the structure of the visual stimulus could therefore engage different brain structures in the task of resolving the perceptual ambiguity. Arguably, the effects of bipolar disorder may be manifest in some brain structures and not others. In order to test this hypothesis further, future experiments should compare switch rates of the same groups of bipolar and control participants for SFM and rivalrous stimuli.

Another striking result was the long perceptual duration for the bistable SFM cylinder for both bipolar participants and controls. Many papers report normalized switch rates rather than actual means, but the latter can often be inferred from other data presented. For the Necker cube, mean perceptual durations have typically been reported between 1 and 3.3 s, and for rivalry between 1 and 10.7 s (Hunt & Guildford 1933; Logothetis *et al*. 1996; Pettigrew & Miller 1998; Brascamp *et al*. 2005; Haynes *et al*. 2005; Lankheet 2006). The length of percept durations can be affected by stimulus properties, drugs and attentional states (George 1936; Carter & Pettigrew 2003; Meng & Tong 2004; van Ee 2005). However, why switch rates are different between paradigms remains unclear.

### (b) Factors affecting switch rate

Many incidental factors influence the rate at which perception switches. We have been able to test throughout our experiment whether participants actually applied focal attention to the task. This was only possible because there are ambiguous and unambiguous versions of rotating SFM figures that are indistinguishable (Nawrot & Blake 1993). Screening participants for attention to the task, by measuring their success in correctly responding to unambiguous stimuli, reduced the perceptual switch rate.

Another finding was that participants with high stereo thresholds showed lower switch rates. This might also be the underlying reason why differences in participants' age would affect switch rate because stereo threshold rises with age (Zaroff *et al*. 2003). Neither Pettigrew & Miller (1998) nor Hunt & Guildford (1933) used age-matched participants in the experimental and control groups. This could have contributed to the differences they find, especially because the participants in control groups tend to be younger (see Miller *et al*. 2003).

A recent paper found that mood could affect the perceptual switch rate for rivalry in participants without psychological disorder (Sheppard & Pettigrew 2006). All participants in our study were euthymic at the time of testing, as assessed with standard psychological tests. Pettigrew & Miller (1998) also stated that the bipolar participants in their study were euthymic at the time of testing, but they do not describe how this was assessed. Differences in participants' mood may be important and the issue needs further exploration, in particular with respect to those with a proven history of bipolar disorder.

In addition to all the factors that we analysed, drug regimes were too diverse to be included in any meaningful comparisons. Here, the study of Hunt & Guildford (1933) offers the unique advantage from the present-day perspective of having tested individuals at a time before the development of modern medications for the treatment of bipolar disorder. Nonetheless, within our study we were able to use modern psychometric methods to establish that all the bipolar patients were euthymic at the time of testing.

### (c) Brain states for perceptual switching

Although the contribution of the above effects is important, it seems improbable that methodological considerations can dismiss all previous reports of differences in perceptual switch times between individuals with bipolar disorder and control groups. Besides this, we also found a small but statistically significant effect consistent with previous reports. The deficits for bipolar patients at the level of perceptual processing are subtler and more specific than those assumed by Pettigrew (2001). However, we did not examine whether switch rates for the SFM cylinder and rivalry might covary from individual to individual. Such a covariation might be attributed to a common oscillator (Carter & Pettigrew 2003) but equally they might be due to the variation in low-level visual capacities, such as stereo threshold, or due to variations in mood (Sheppard & Pettigrew 2006).

In terms of the brain structures responsible for these simple cognitive decisions, it is improbable that a single brain region functions as a switch driving perceptual changes in SFM and all other rivalrous figures. Visual psychophysics shows that small changes in sensory input can affect perceptual alternations (Blake *et al*. 2003; Brouwer & van Ee 2006), suggesting a role for local circuits within visual cortex. The capacity of neuronal circuits to generate switches in the perceptual interpretation of figures is certainly widespread (Logothetis & Schall 1989; Leopold & Logothetis 1996; Brouwer & van Ee 2007), probably reflecting elements of cortical circuitry which exist in common in diverse parts of the neocortex. In this context, the similar effect of lysergic acid diethylamide (LSD) on different forms of perceptual rivalry has been advanced as an argument in favour of a single site governing rivalry (Carter & Pettigrew 2003). However, an alternative explanation is the involvement of similar neurotransmitters in the same pattern of neuronal circuitry in diverse anatomical locations. Although local cortical circuits clearly receive some attention-related influences from the prefrontal cortex (Windmann *et al*. 2006) and it has been shown that activity in inferior frontal cortex precedes rivalrous switches (Sterzer & Kleinschmidt 2007), the local structure and dynamics of the cortical circuitry are a major factor in setting up perceptual instability (Parker & Krug 2003).

## Supplementary Material

Further descriptions and references

Stimulus illustration The included movie illustrates the general type of ambiguous stimulus used for these experiments. The attached movie does not replicate all aspects of the stimulus used in the experiments. In particular, the lifetime of the dots as viewed in a movie loop is infinite, whereas for the stimuli used in the experiments, individual dots in the display had a limited lifetime (see Methods of the main paper). It is also impossible to replicate the viewing conditions experienced by the participants by inspecting the movie on a computer after downloading this movie file from the internet

Figure 1 The two experimental set-ups

Figure 2 Comparison of performance on anaglyph stereo display and Wheatstone stereo display

Figure 3 Comparison of fitting parameters for gamma and gamma-rate fits

Figure 4 The cumulative distribution function (CDF) for participants with good performance (catch events correct≥75%)
