# Supplementary material for: Perceptual switch rates with ambiguous structure-from-motion figures in bipolar disorder
Source: Proc Biol Sci. 2008 May 7;275(1645):1839–48. doi: 10.1098/rspb.2008.0043 (PMC2494571; doi:10.1098/rspb.2008.0043)
Supplement: Supplementary file 1 [file rspb20080043s04.pdf]

(a)

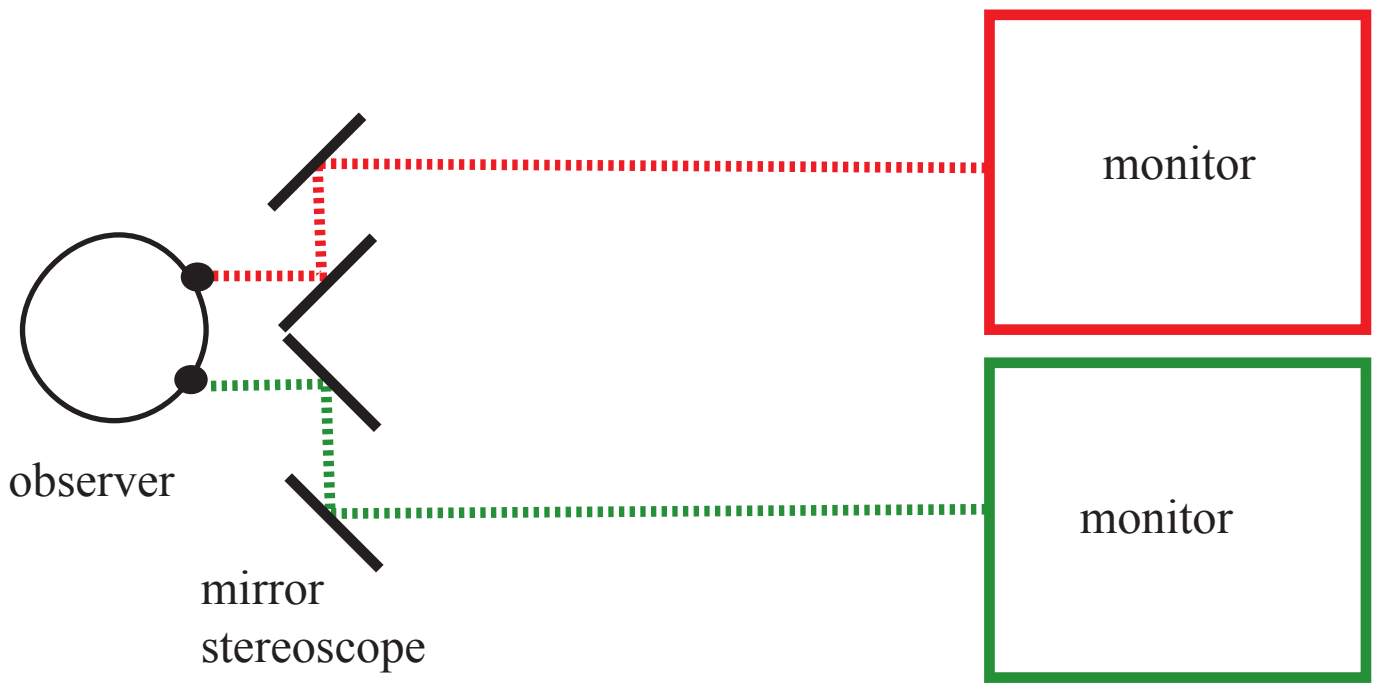
monitor
monitor
observer
mirror
stereoscope

(b)

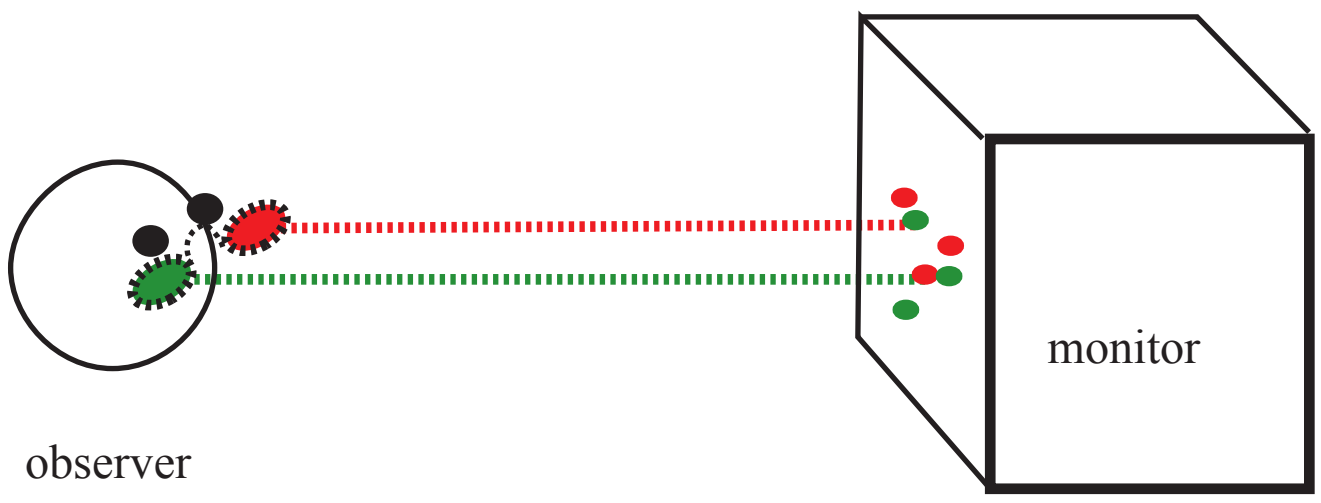
monitor
observer
