# Supplementary material for: Perceptual switch rates with ambiguous structure-from-motion figures in bipolar disorder
Source: Proc Biol Sci. 2008 May 7;275(1645):1839–48. doi: 10.1098/rspb.2008.0043 (PMC2494571; doi:10.1098/rspb.2008.0043)

(a) all control participants

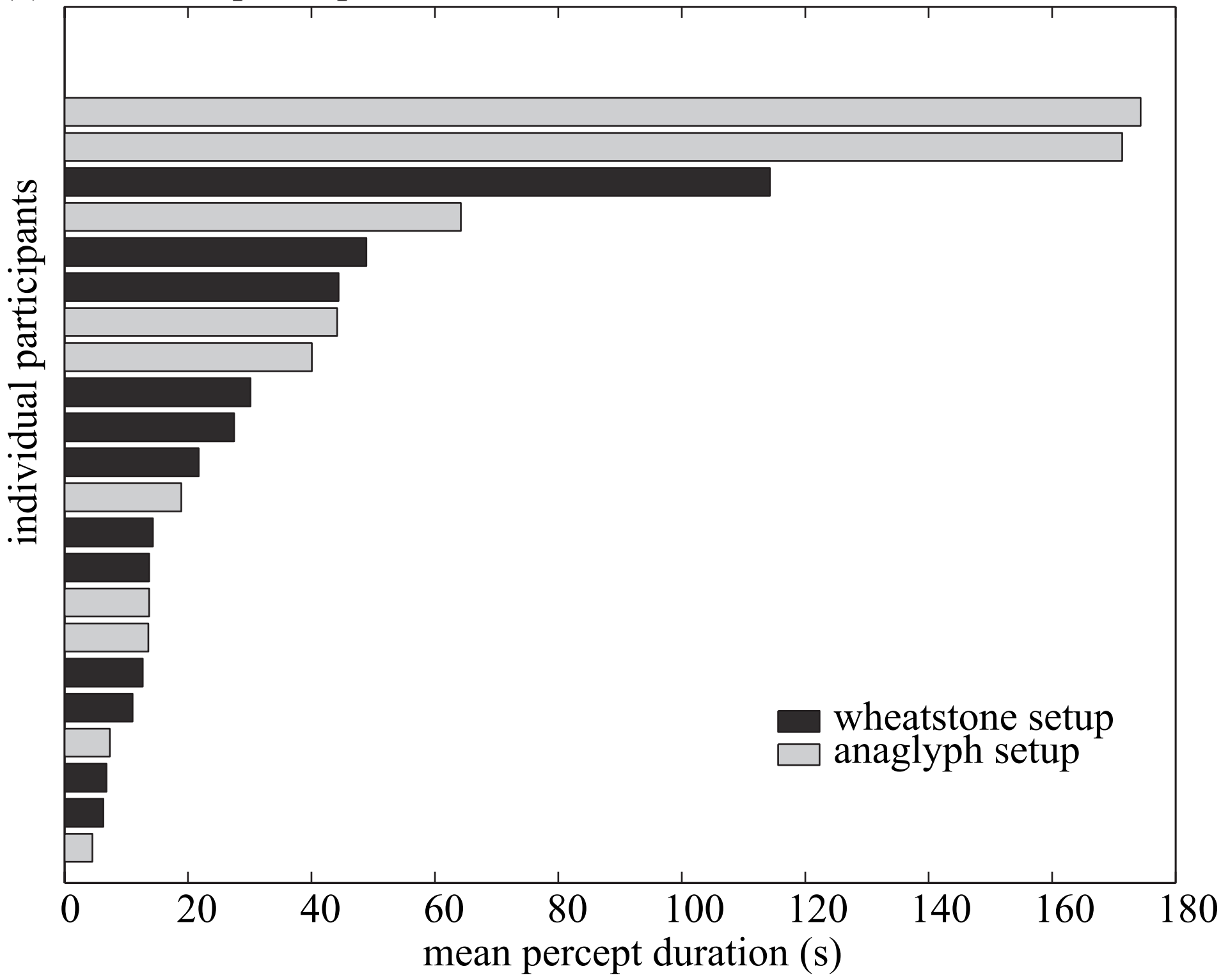

(b) control participants >= 75% correct for catch events

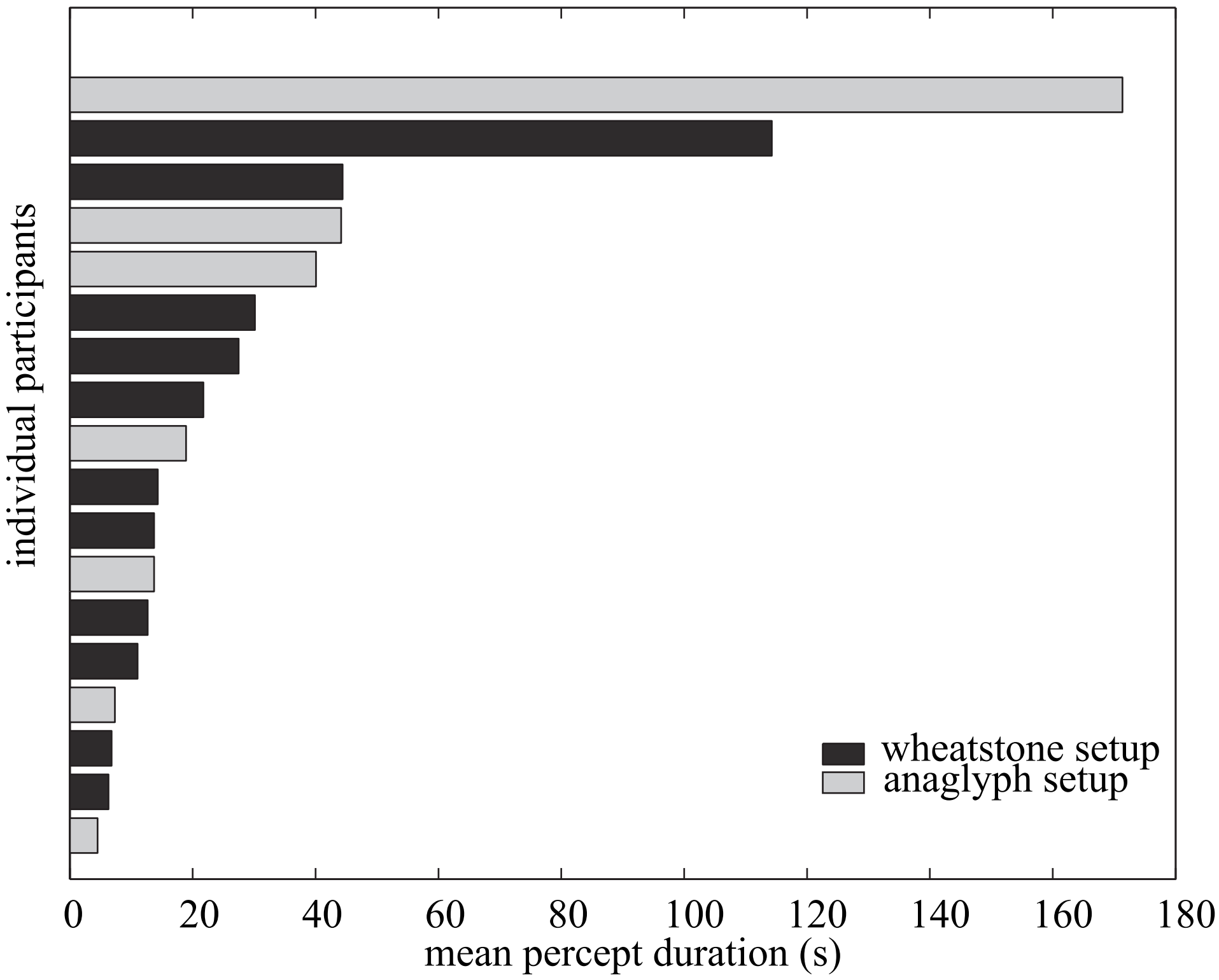

Supplement: Supplementary file 2 [file rspb20080043s05.pdf]
