# Supplementary material for: Perceptual switch rates with ambiguous structure-from-motion figures in bipolar disorder
Source: Proc Biol Sci. 2008 May 7;275(1645):1839–48. doi: 10.1098/rspb.2008.0043 (PMC2494571; doi:10.1098/rspb.2008.0043)

participants with 75% or more of catch events correct

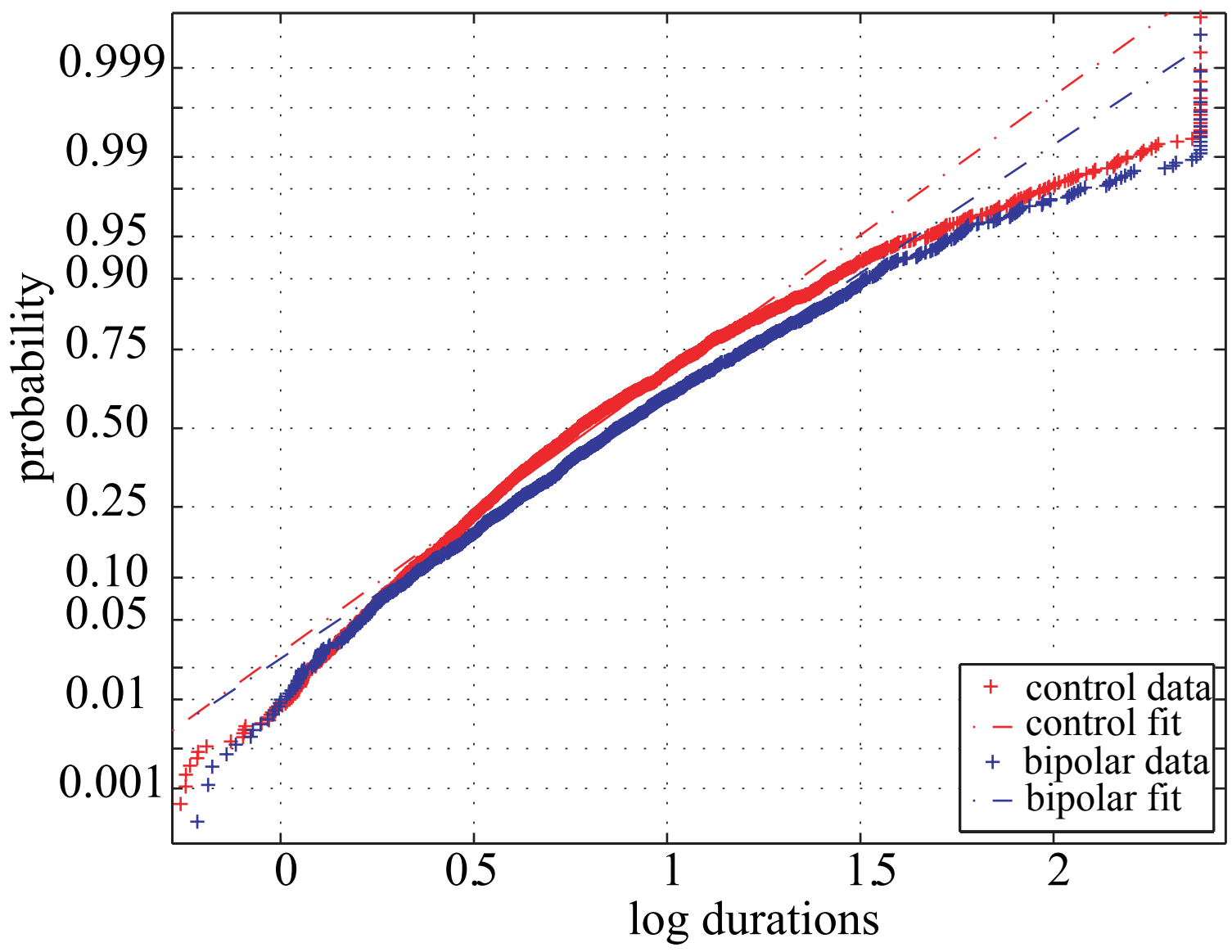

Supplement: Supplementary file 4 [file rspb20080043s07.pdf]
