# Supplementary material for: Perceptual switch rates with ambiguous structure-from-motion figures in bipolar disorder
Source: Proc Biol Sci. 2008 May 7;275(1645):1839–48. doi: 10.1098/rspb.2008.0043 (PMC2494571; doi:10.1098/rspb.2008.0043)
Supplement: Supplementary file 5 [file rspb20080043s02.doc]

ON-LINE SUPPLEMENTARY MATERIAL

**Movie: Stimulus Illustration.**

The included movie illustrates the general type of ambiguous stimulus used for these experiments. The attached movie does not replicate all aspects of the stimulus used in the experiments. In particular, the lifetime of the dots as viewed in a movie loop is infinite, whereas for the stimuli used in the experiments, individual dots in the display had a limited lifetime (see Methods of the main paper). It is also impossible to replicate the viewing conditions experienced by the participants by inspecting the movie on a computer after downloading this movie file from the internet.

**Factors affecting Cylinder Perception**

*Temporal Sequence of Responses*

We examined the pattern of percept durations for each participant in order to establish whether the persistence of a percept was dependent on preceding percept durations. Autocorrelation coefficients were calculated for each participant. These coefficients measure whether there was any association between percept durations, such that, for example, a long duration is followed by a short duration for the alternate percept. If the percept durations are completely uncorrelated then for a large sample size (N), the correlation coefficient should follow approximately a normal distribution with a mean of 0 and a variance of 1/N. Autocorrelations were evaluated as to whether they were within the 95% or 99% confidence interval of the expected value of an autocorrelation coefficient that was truly random and distributed normally (α=0.05 and α=0.01 level; two-tailed tests). Of the participants with at least 10 or more data points for 1 or more lags (coefficients for 1-4 lags were computed), only 4/13 controls and 6/12 patients had a autocorrelation coefficient where the null hypothesis of no serial correlation was rejected at the α=0.05 level, and 3/13 controls and 4/12 patients had autocorrelation coefficients that were significant at the α=0.01 level. We also examined the data with a Lathrop statistic (Logothetis et al. 1996). Of the 19 control participants and 17 patients with enough data to perform this analysis, only one control had a Lathrop statistic significantly deviating from 1 (p=0.042).

The analysis of autocorrelation coefficients and Lathrop statistics suggest that for the majority of individuals in this study, the duration of a percept was generally not dependent on the duration of the preceding percept, in agreement with previous reports (Fox & Herrmann 1967; Logothetis et al. 1996). Both of these analyses established that any serial dependency in percept durations is weak and, importantly for this paper, the bipolar and control groups of participants did not differ from one another in this regard.

Although the analysis of autocorrelation coefficients indicated that there was little interaction between successive viewing periods, we also wondered whether participants might be systematically biased towards one perceptual interpretation of the ambiguous figure. On this argument, the perceptual switch rates would be slow because the participants are almost permanently “stuck” in just one of the two perceptual states. Both patients and controls showed a tendency towards viewing the ambiguous stimulus as rotating clockwise (dots in front surface moving left) rather than counterclockwise (dots in front surface moving right). On average, the ratio of durations for which the ambiguous stimulus was viewed as rotating counterclockwise, as opposed to clockwise, was 0.82 for controls and 0.61 for patients (with standard deviations of 0.51 and 0.32 respectively). A ratio of ‘1’ would be perfectly balanced. If one includes only participants who successfully detected at least 75% of the catch trials, this gives ratios of 0.88 for controls and 0.58 for patients. None of these differences between the patients and the controls was significant (Mann-Whitney test, p>0.15 for all participants, p>0.71 for the participants that passed the catch events).

*Alternative perceptual interpretations.*

Hol and colleagues (2003) have pointed out that alternative perceptual interpretations of ambiguous structure-from-motion stimuli are possible. In their study, participants reported a ‘two-shell percept’, still at different perceived depths but of the same curvature. Our stimuli differed in various ways from the one used in the study by Hol and colleagues: for instance, in our study dots had a limited life-time and left and rightwards moving dots could have the same horizontal trajectory. None of our participants reported seeing a shell-percept. However, in the event that participants had very occasionally perceived ‘the two-shells’, this would have been unlikely to affect the results in the present study, because the moving dot surfaces would still have had a distinct depth order.

*Eye movements*

In this study, we did not set out to investigate whether different patterns of eye movements affect our measurement of perceptual durations. Eye position has been shown to change around the time of perceptual switches for the Necker cube (Einhauser et al. 2004). Also, binocular pursuit eye movements can follow the perceived depth in ambiguous SFM figures (Ringach et al. 1996). However, early work, using the technique of paralysing human eye muscles, indicated that eye movements are not necessary to generate bistability (George 1936). Eye movements have also been examined for monkeys during perceptual judgements of a SFM stimulus, similar to the one used in this paper (Dodd et al. 2001). This analysis has shown that eye movements are not directly responsible for the reported perceptual choice. That saccades and smooth pursuit eye movements are not essential for perceptual alternations in ambiguous SFM figures has recently been confirmed in human participants (Brouwer & van Ee 2006).

**Supplementary Figure 1. The two experimental set-ups.**

The anaglyph system is not only more portable but also easier to use for naïve participants. Therefore it was considered the more appropriate choice for the patient group and more robust in a clinical setting. On the other hand, a well-aligned mirror system offers better-matched images with less optical distortion.

(a) In the Wheatstone stereoscope, participants viewed separate monitors with each eye through two sets of mirrors. This allowed us to introduce binocular disparity between the images presented to the left and right eye. (b) In the anaglyph set-up, participants viewed a single monitor through red-green glasses. Binocular disparity was introduced by displaying the stimulus for one eye in red and for the other eye in green. The dot colours had been calibrated to minimize leakage through the colour filters of the glasses.

**Supplementary Figure 2. Comparison of performance on anaglyph stereo display and Wheatstone stereo display.**

All bipolar patients were tested with the anaglyphic set-up. Control participants were either tested on a Wheatstone stereo display (n=12; mean duration 29.31s) or on an anaglyphic display viewed with red-green glasses (n=10; mean percept duration 55.22s). (a) A Mann-Whitney test revealed no statistically significant difference between the percept durations of the two control groups (p>0.53). (b) A similar result was obtained when participants were included only if they obtained correctly at least 75% of the catch events (Wheatstone 39.19s; anaglyphic display 31.58s; p>0.61). Since there was no difference between the percept durations measured in the two set-ups, in this paper we combined the data.

**Supplementary Table 1: Fitting the data for each individual participant.**

|  | Gamma fit | Log Normal fit | Gamma rate fit |
| --- | --- | --- | --- |
| control  participants | 8/13 | 11/13 | 11/16 |
| bipolar  participants | 6/14 | 10/13 | 8/14 |

A distribution X was considered to provide a good fit of an individual participant’s data, if a chi-square goodness of fit test could not reject the null hypothesis (at the 5% significance level) that distribution X had generated the individual’s data. Note that this test requires specifying bin sizes, such that there are at least 5 expected counts per bin: some participants had so few data points overall that there were insufficient bins with greater than 5 counts to run the chi-square goodness of fit test, with the result that data from these participants could not be evaluated by this approach. Bins were picked automatically, not by hand. We conclude from this analysis that there is no single distribution that could provide a satisfactory fit to the data from all participants, although the log-normal distribution does provide satisfactory fits to the data from more participants than any other distribution.

**Supplementary Figure 3. Comparison of fitting parameters for gamma and gamma-rate fits.**

Switch rates were fitted separately for each participant with the best fitting gamma-rate distribution (a) and percept durations with the best fitting gamma distribution (b). The two fitting parameters for each individual and each distribution are plotted against each other in these figures. Open circles represent the parameters from bipolar participants, closed diamonds the control data. There is no segregation between the parameters for the two groups of participants in either distribution.

**Supplementary Figure 4. The cumulative distribution function (CDF) for participants with good performance (catch events correct >=75%).**

The CDF for pooled participant percept durations was plotted on a log scale. Patient log percept durations (blue) and control log percept durations (red) were pooled separately after normalization. The pooled patient and pooled control data roughly follow a straight line, indicating that after transformation, the data are approximately normally distributed. The curve for the control participants rises faster indicating that the control group has shorter percept durations than the patient group. The best fitting line for the control data could not adequately describe the patient data (χ^2^=37, df=2, p<0.001).

**References**

Brouwer, G. J. & van Ee, R. 2006. Endogenous influences on perceptual bistability depend on exogenous stimulus characteristics. Vision Res. 46, 3393-3402.

Dodd J. V., Krug, K., Cumming, B. G. & Parker, A. J. 2001 Perceptually bistable three-dimensional figures evoke high choice probabilities in cortical area MT. J. Neurosci. 21, 4809-4821.

Einhauser W., Martin K. A. & Konig P. 2004 Are switches in perception of the Necker cube related to eye position? Eur. J. Neurosci. 20, 2811-2818.

Fox, R. & Herrmann, J. 1967 Stochastic properties of binocular rivalry alternations. Perception & Psychophysics 2, 432-36.

George, R. W. 1936 The significance of the fluctuations experienced in observing ambiguous figures and in binocular rivalry. J. Gen. Psych. 15, 39-61.

Hol, K., Koene, A. & van Ee, R. V. 2003 Attention-biased multi-stable surface perception in three-dimensional structure-from-motion. J. Vision 3, 486-498

Logothetis, N. K., Leopold, D. A. & Sheinberg, D. L. 1996 What is rivalling during binocular rivalry? Nature 380, 621-624.

Ringach, D. L., Hawken, M. J. & Shapley, R. 1996 Binocular eye movements caused by the Perception of Three-Dimensional structure from motion. Vision Res. 36, 1479-1492.
